# Synergistic Effects of Salt Concentration and Working Temperature towards Dendrite-Free Lithium Deposition

**DOI:** 10.34133/2019/7481319

**Published:** 2019-11-05

**Authors:** Panlong Li, Chao Li, Yang Yang, Chanyuan Zhang, Renhe Wang, Yao Liu, Yonggang Wang, Jiayan Luo, Xiaoli Dong, Yongyao Xia

**Affiliations:** ^1^Department of Chemistry and Shanghai Key Laboratory of Molecular Catalysis and Innovative Materials, Institute of New Energy, Fudan University, Shanghai 200433, China; ^2^State Key Laboratory of Chemistry Engineering, School of Chemical Engineering and Technology, Tianjin University, Tianjin 300072, China

## Abstract

The lithium- (Li-) metal anode is crucial for developing high-energy-density batteries, while its dendritic growth and the low charge/discharge Coulombic efficiency in organic electrolytes hinder its practical application. Herein, we employed an in situ optical microscope to investigate the effect of the electrolyte concentration and the working temperature on the Li-plating/-stripping process. It is found that a higher concentration electrolyte can suppress its side reaction to improve the charge/discharge Coulombic efficiency, and a higher temperature can help lithium plate/strip uniformly with less lithium dendritic growth. An average Coulombic efficiency was obtained as high as 99.2% for over 150 cycles with a fixed plating capacity of 2 mAh cm^−2^ on copper foil in a 3 mol/kg ether-based electrolyte under 60°C, which provides an efficient and facile strategy for developing high-performance Li-metal batteries.

## 1. Introduction

Li-metal anode has been regarded as one of the most promising candidates for next-generation high-energy-density batteries, owing to its ultra-high capacity and low potential [1–3]. Unfortunately, metallic Li shows hyperactivity with various organic solvents, leading to side reactions with commonly used electrolytes [4, 5]. It was widely reported that these reactions could form a solid electrolyte interface (SEI) layer to prevent further anode corrosion [6]. However, once uncontrollable dendrite morphology is formed during long cycles, numerous fractures or cracks will occur in the existing SEI. The newly exposed Li will further consume the electrolyte to form a new SEI film, resulting in low Coulombic efficiency (CE) [7]. Moreover, the uncontrolled dendritic Li metal will cause internal short circuit, even severe safety issues [8].

High CE and dendrite-free behaviour of Li deposition under deep plating/stripping are essential for the practical application of the Li-metal anode [7]. Considerable approaches [9–13] and technologies [14–19] have been focused on achieving these two targets. As an indispensable component of batteries, the electrolyte has been deeply investigated to improve Li deposition behaviour, including modifying solvents [20, 21], additives [22, 23], and lithium salts [5, 24, 25]. Towards organic solvents, glymes are reported to be less reactive with Li than cyclic ethers, esters, and alkyl carbonates [5]. However, there are still side reactions occurring between glymes and the Li-metal anode, resulting in low CE. Recently, applying highly concentrated electrolytes are demonstrated to be an effective strategy to achieve high CE and regulate Li deposition morphology under deep Li-plating/-stripping, because increasing salt concentration is in favour of forming a more stable SEI layer, so as to suppress further reactions with metallic Li [5, 24, 26, 27]. Unfortunately, highly concentrated electrolytes could not fully restrain dendrite Li growth. Even worse, they showed high viscosity and low ionic conductivity, resulting in large nucleation overpotential and internal resistance [28–30].

Apart from electrolytes, temperature is usually regarded as an extrinsic factor to electrochemical reaction kinetics [31], which is seldom studied in Li dendrite formation. Koratkar's group [32] proposed that self-heating could heal Li dendrites by applying high current density (15 mA cm^−2^), because the mass transport rate is related to the temperature. The stepped-up Li^+^ distribution will heal the dendrites and simultaneously smoothen the surface of Li anodes [32]. However, rising temperature will accelerate the reaction of Li metal with electrolytes, which leads to irreversible capacity fading. Hence, more work remains for developing high-energy Li-metal batteries which are dendrite-free and with high CE.

In this work, we propose a high CE and dendrite-free Li deposition behaviour, accomplished through the synergistic effects of electrolyte concentration and working temperature. Highly concentrated electrolytes can effectively increase the CE, and rising temperature helps to guide the homogeneous Li deposition under deep Li-plating/-stripping. In addition, elevated temperature and high-salt concentration contribute to form a stable LiF-rich SEI film, which further suppresses the lithium dendrite growth. As a result, the goals of high average CE and dendrite-free Li deposition are achieved under the optimized condition (3m electrolyte at 60°C). The mechanism of the synergistic effect is revealed through a series of in situ optical microscopy, ex situ scanning electron microscopy (SEM), and X-ray photoelectron spectroscopy (XPS) characterizations.

## 2. Results

### 2.1. Optimization of Electrolyte and Temperature

**Figure 1:**
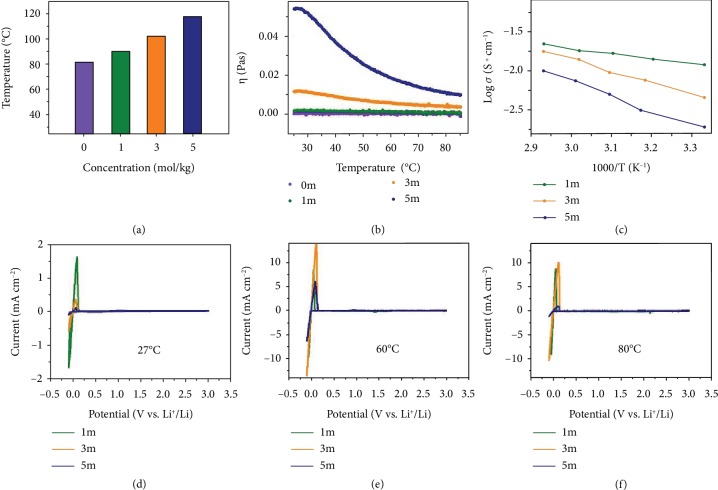
Physicochemical properties of different concentration electrolytes. (a) The boiling point of pure solvents (DOL : DME = 1 : 1 by volume), 1m, 3m, and 5m electrolytes. (b) The curves of viscosity and temperature for pure solvents, 1m, 3m, and 5m electrolytes. (c) Arrhenius plots of 1m, 3m, and 5m electrolytes. The cyclic voltammograms of 1m, 3m, and 5m electrolytes at (d) 27°C, (e) 60°C, and (f) 80°C.

Ether-based electrolytes with different concentrations were prepared by adding 1 mol/kg (1m), 3 mol/kg (3m), and 5 mol/kg (5m) lithium bis(trifluoromethanesulfonyl)imide (LiN(SO_2_CF_3_)_2_ or LiTFSI) into 1 : 1 vol/vol 1,3-dioxolane/1,2-dimethoxyethane (DOL/DME) with 1% lithium nitrate (LiNO_3_) (by weight) additive (termed 1m, 3m, and 5m electrolytes, respectively). The boiling point of the liquid electrolyte was studied to determine suitable working temperature for safety concerns. High-salt concentration increases the boiling point of the liquid electrolyte, which ensures a wider temperature range for safe operation (Figure 1(a)). Compared with the boiling point of 1 : 1 vol/vol DOL/DME solvents (81.1°C), those of 1m, 3m, and 5m electrolytes are 88.9°C, 101.4°C, and 117.8°C, respectively. Unfortunately, high-salt concentration magnifies the viscosity of the liquid electrolyte, which slows down the speed of ion transportation and augments the problem of concentration polarization. This will inevitably affect the electrochemical performance of lithium metal deposition such as high polarization voltage and lithium dendrite growth [20]. As shown in Figure 1(b), the viscosity of highly concentrated electrolytes (3m and 5m electrolytes) is much higher than that of the 1m electrolyte at room temperature (27°C). After raising the temperature, the viscosity of highly concentrated electrolytes is reduced dramatically. The viscosity of the 3m electrolyte above 60°C is similar to that of the 1m electrolyte at room temperature. The viscosity of the 5m electrolyte above 80°C is approximate to that of the 3m electrolyte at room temperature (27°C) but is still higher than that of the 1m electrolyte at room temperature (Figure 1(b)).

For another, the ionic conductivity of different concentrated electrolytes was measured in a symmetric Pt|electrolyte|Pt cell by AC impedance spectroscopy. As shown in Figure 1(c) and [Supplementary-material supplementary-material-1], the ionic conductivity of electrolytes increases with the temperature. The ionic conductivity of the 3m electrolyte at 60°C is 14.0 mS cm^−1^, which is close to that of the 1m electrolyte at 27°C (11.9 mS cm^−1^). In spite of the elevated temperature (60°C), the higher concentration of the 5m electrolyte still shows a little lower ionic conductivity (7.4 mS cm^−1^), owing to its high viscosity. With further increase of the temperature to 80°C, the 5m electrolyte behaves in a similar ionic conductivity of 12.7 mS cm^−1^. This is because ionic conductivity is proportional to the number of mobile ions and inversely proportional to the viscosity [29].

Moreover, Li-plating/-stripping behaviours in different concentrated electrolytes at various working temperatures were assessed systematically by cyclic voltammetry. As presented in Figure 1(d)–1(f), reversible Li-plating/-stripping can be detected in the three kinds of electrolytes. At 27°C, it showed higher peak current density in the 1m electrolyte than in 3m and 5m (Figure 1(d)), which is attributed to its higher conductivity and lower viscosity. Benefitting from the elevated temperature (60°C and 80°C), the peak current density in concentrated electrolytes (3m and 5m) is evidently improved at 60°C and 80°C, which are close to that of the 1m electrolyte (Figures 1(e) and 1(f)). Notably, proper viscosity and higher Li^+^ concentration enabled the 3m electrolyte with the highest peak current density at 60°C, proving the effect of increasing the concentration and raising the temperature. The results also preliminarily indicated the synergistic effect of the concentration and temperature.

### 2.2. Performance of Li-Plating and Li-Stripping on Copper (Cu) Foil

**Figure 2:**
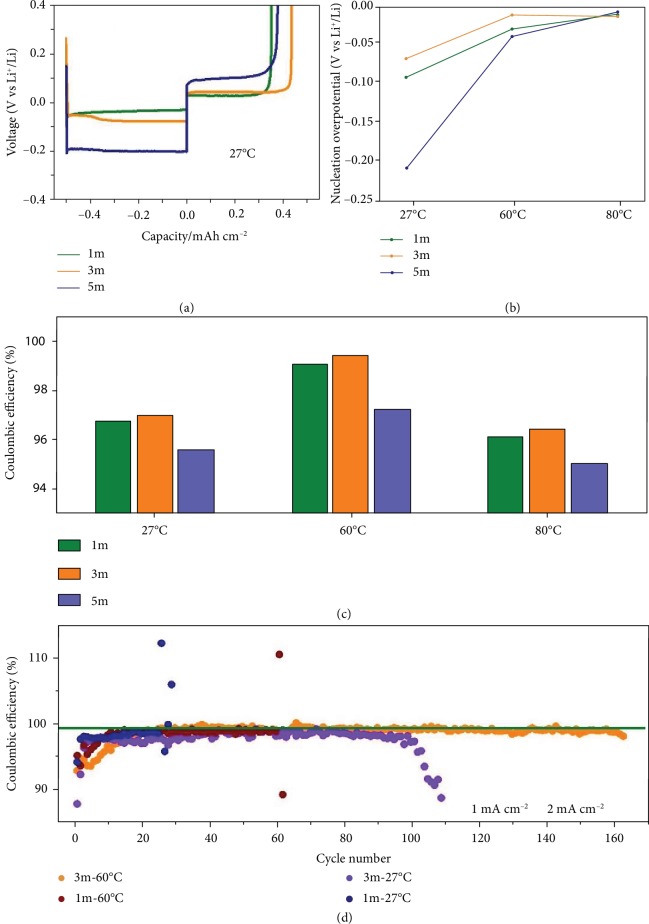
The investigation of the Li-plating and Li-stripping process on copper foil in Li/Cu half cells. (a) The initial polarization curves of the plating/stripping behaviour of Li on copper foil at the current density of 0.5 mA cm^−2^ with a fixed capacity of 0.5 mA h cm^−2^ in 1m, 3m, and 5m electrolytes at 27°C. (b) The nucleation overpotential values under different salt concentrations and working temperatures. (c) Average CE of the first 100 stable cycles at the current density of 0.5 mA cm^−2^ with a fixed capacity of 0.5 mAh cm^−2^ under the condition of different concentration electrolytes (1m, 3m, and 5m) and temperatures (27°C, 60°C, and 80°C). (d) The CE of the Li-plating/-stripping process on copper foil at the current density of 1 mA cm^−2^ with a fixed capacity of 2 mAh cm^−2^ in 1m and 3m electrolytes at 27°C and 60°C.

To elucidate the synergistic effects of salt concentration and working temperature, the performance of Li-plating/-stripping on Cu foil was investigated under nine different conditions (three kinds of salt concentrations and three kinds of working temperatures). The Li/Cu half cells were operated at the current density of 0.5 mA cm^−2^ with a fixed capacity of 0.5 mAh cm^−2^. The voltage profiles of plating/stripping processes at 27°C in Figure 2(a) reflect the influence of concentration. The 5m electrolyte shows huge polarization compared to that of the 1m and 3m electrolytes, attributing to the large viscosity of the high-salt concentration. Raising the working temperature evidently facilitates to reduce the high viscosity of the electrolyte, so as to decrease the stable platform ([Supplementary-material supplementary-material-1]). Nucleation overpotential is related to the metallic Li nucleation process, which causes the metal to distribute uniformly on the anode surface [35]. The relationship of nucleation overpotential with concentration and temperature is plotted as in Figure 2(b), from which the 3m electrolyte behaves with the smallest overpotential at 60°C. It can be detected that the nucleation process became easier at elevated temperature. This is because mass transport can be sped up with the increase of temperature. But too high a temperature would also bring up severe chemical instability of the electrolyte with the Li metal. Although fresh Li foil showed excellent stability with the 3m electrolyte at 60°C for 30 days ([Supplementary-material supplementary-material-1]), it was visibly darkening and the electrolyte became turbid after merely 1 day at 80°C ([Supplementary-material supplementary-material-1]). After 30 days in the 3m electrolyte at 80°C, the surface of the Li foil was thoroughly blackened ([Supplementary-material supplementary-material-1]), indicating the shortcoming caused by too high a temperature.

High CE is another important factor to assess the Li-plating/-stripping behaviour. Average CEs of the first 100 cycles for the nine different conditions were calculated and compared in Figure 2(c). The applied current density and capacity were 0.5 mA cm^−2^ and 0.5 mAh cm^−2^, respectively. Amongst these conditions, the 3m electrolyte shows outstanding CE at 60°C, indicating the optimal effect of concentration and temperature. To further investigate the CEs at higher current density and higher deposited capacity, low viscosity electrolytes (1m and 3m) at 27°C and 60°C were selected to make a comparison. As shown in Figure 2(d), the cells in the 1m electrolyte at 27°C and 60°C can only stay stable for less than 70 cycles at the current density of 1 mA cm^−2^ with a fixed capacity of 2 mAh cm^−2^ due to the short circuit caused by dendrite formation. With optimized salt concentration (3m) and temperature (60°C), the cells can keep over 150 stable cycles with high average CE of 99.2%, which is much better than those at room temperature. The corresponding charge/discharge profiles of half cells are given in [Supplementary-material supplementary-material-1]. Li-plating/-stripping performance was also investigated using Li/Li@Cu symmetric cells at 27°C and 60°C in 1m and 3m electrolytes. As shown in [Supplementary-material supplementary-material-1], these symmetric cells in the 3m electrolyte at 60°C deliver the lowest overpotential and longest lifetime (over 1200 hours) among these four conditions. Meanwhile, Li@Cu/LiFePO_4_ cells tests were carried out to demonstrate the influence of temperatures and concentrations of electrolytes, which is shown in [Supplementary-material supplementary-material-1]. Those cells in the 3m electrolyte at 60°C show the best cycling performance and lowest overpotential among four kinds of cells in different conditions. These results can well demonstrate the synergistic effects of concentrated electrolyte and elevated temperature on the high performance of Li deposition behaviour such as long lifespan and low overpotential.

### 2.3. Investigation of Li Dendrite Evolution by In Situ Optical Microscopy

**Figure 3:**
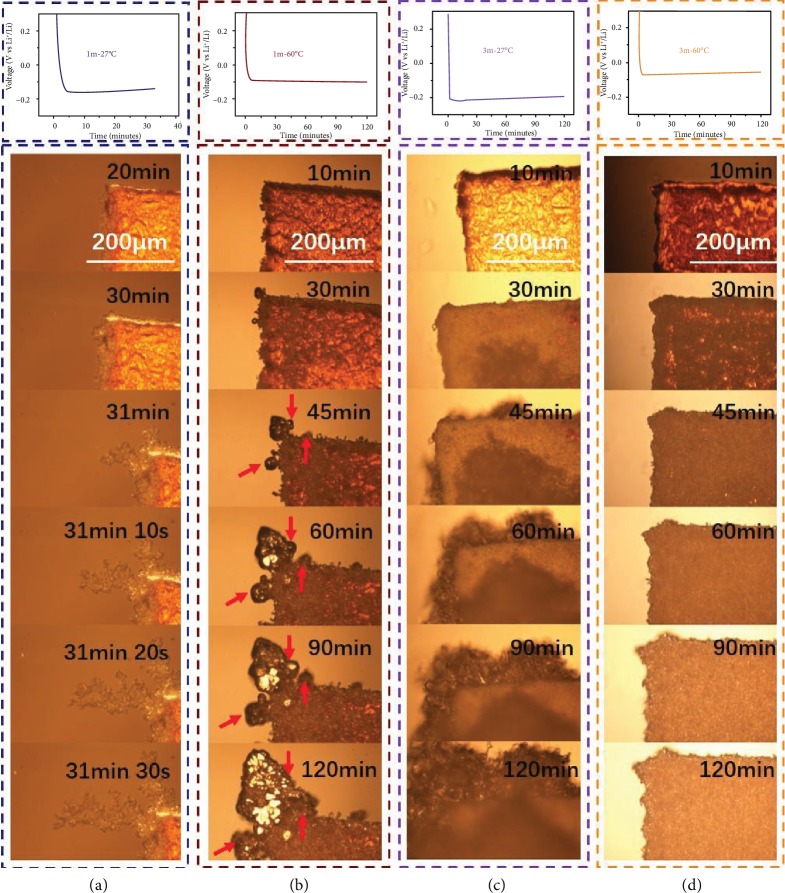
Voltage profiles and the corresponding in situ optical images of Li deposition morphologies under different conditions. (a) The voltage profiles of in situ cells and deposition morphologies of Li in 1m electrolyte at room temperature. After half an hour, Li dendrites grew up explosively, just like a tree branch. (b) The voltage profiles of in situ cells and deposition morphologies of Li in 1m electrolyte at 60°C. The explosive growth of Li dendrites was greatly suppressed, and three dendrites fused into one gradually (the red arrows). (c) The voltage profiles of in situ cells and deposition morphologies of Li in 3m electrolyte at room temperature. The anode surface was rough and coarse. (d) The voltage profiles of in situ cells and deposition morphologies of Li in 3m electrolyte at 60°C. The anode surface was dense and smooth without Li dendrites.

To obtain a better understanding of the dendrite nucleation and growth process, in situ observation was carried out through an optical microscope (Figure 3 and [Supplementary-material supplementary-material-1]). As presented in Figure 3, the electrochemical overpotential increased with the increase of concentration (1m vs. 3m) and decreased with the increase of temperature (27°C vs. 60°C). In turn, distinct growth patterns emerge with various concentrations and temperatures. When working at a low concentration of the 1m electrolyte and temperature of 27°C (Figure 3(a)), dendrites spring out in the initial 30 min and grow into a branch-like structure rapidly ([Supplementary-material supplementary-material-1]). Increasing the temperature to 60°C (Figure 3(b)) can relieve the dendritic structure because the motivated diffusion of Li enables the fusion of the dendrites under high temperature [32]. It can be detected from Figure 3(c) that a different morphology arose when a high concentration (3m) was applied. A large number of tiny branches appeared with longer times of the deposition process, which can be attributed to more nucleation sites in the concentrated electrolyte. Benefitting from high concentration and elevated temperature, there are almost no obvious dendrites through the entire deposition procedure in the 3m electrolyte at 60°C (Figure 3(d)), which evaluated well the synergetic effect of concentration and temperature.

Furthermore, in situ optical observation of Li-plating behaviour was also conducted in LB303 (i.e., 1 mol/L (1 M) LiPF_6_ in 1 : 1 : 1 vol/vol/vol ethylene carbonate/diethyl carbonate/dimethyl carbonate (EC/DEC/DMC) ([Supplementary-material supplementary-material-1])), in comparison with the 1m electrolyte ([Supplementary-material supplementary-material-1] and [Supplementary-material supplementary-material-1]). The behaviour of the former deposited Li tended to form loose and porous deposits due to the drastic side reactions and fragile SEI layer, while the latter started without Li dendrites. However, once the dendrite formation occurred during cycling, Li dendrites grew up out of control ([Supplementary-material supplementary-material-1] and [Supplementary-material supplementary-material-1]). After using glass fibers as a separator to fabricate in situ cells, Li dendrites were inclined to puncture though the separator according to [Supplementary-material supplementary-material-1], which is possible owing to the bad mechanical and physical performance of glass fibers.

### 2.4. Characterization of Li-Metal Deposition by Ex Situ SEM and Ex Situ XPS

**Figure 4:**
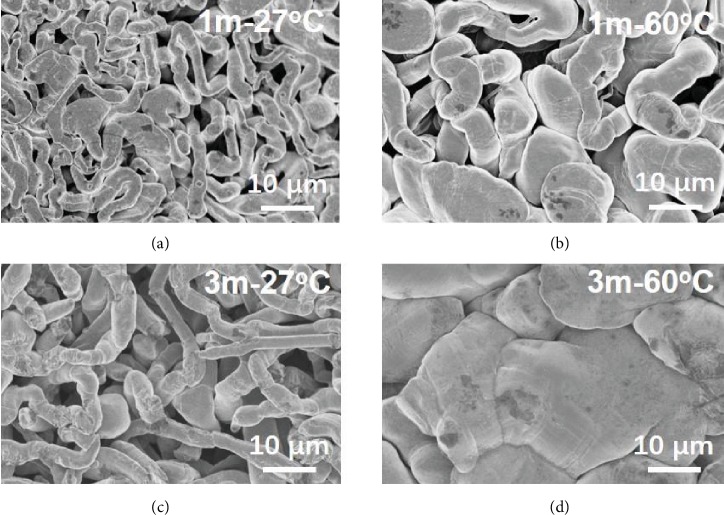
Characterization of Li-metal deposition by ex situ SEM. Li was plated on copper foil at the current density of 1 mA cm^−2^ with a fixed capacity of 2 mAh cm^−2^ in 1m and 3m electrolytes at different temperatures. (a) Top-view SEM images of deposition morphology of Li in 1m electrolyte at 27°C; (b) 1m electrolyte at 60°C; (c) 3m electrolyte at 27°C; (d) 3m electrolyte at 60°C.

Different deposition mechanism leads to various morphologies of deposited Li, which can be revealed with the ex situ SEM characterization, as given in Figure 4. The same procedures with a fixed capacity of 2 mAh cm^−2^ plated on copper foil were conducted on the different electrolytes and temperatures. It can be depicted from Figure 4(a) that a linear and porous structure was obtained in the 1m electrolyte at 27°C, while a bigger size and relatively smooth surface can be detected in same concentration at 60°C (Figure 4(b)). This means that rising temperature enabled deposited Li without the sharp dendritic features, which is in correspondence with the in situ optical observations. Similarly, the deposited Li showed similar porous morphology in the 3m electrolyte at room temperature of 27°C and a relatively flat surface at 60°C (Figures 4(c) and 4(d)). The densely packed and flat deposition indicated the dendrite-free behaviour in the 3m electrolyte at 60°C, which shows a smaller surface area so as to alleviate the side reactions with the electrolyte. This explained well the high CE at the optimal condition (the concentration of the 3m electrolyte and working temperature of 60°C) in Figure 2.

**Figure 5:**
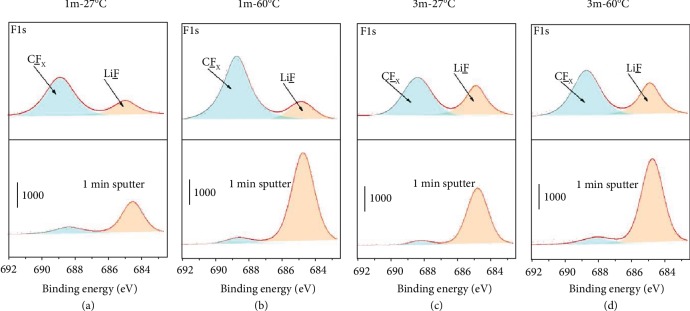
Characterization of the SEI components of deposited Li by XPS. XPS depth profiles of F1s spectra of deposited Li before and after 1 min Ar sputtering in (a) 1m electrolyte at 27°C, (b) 1m electrolyte at 60°C, (c) 3m electrolyte at 27°C, and (d) 3m electrolyte at 60°C.

To further explore the nature of the various deposited Li behaviours, the components of SEI layers were characterized and analysed by X-ray photoelectron spectroscopy (XPS) depth profiles. Ar sputtering was used to remove the impacts of electrode exposure to air. As shown in Figure 5(a)–5(d), the F1s spectrum of four samples under different conditions exhibits two main peaks at ~684.8 eV (attributed to LiF) and 688.8 eV (attributed to CF_x_) [33]. Before Ar sputtering, the samples in the 3m electrolytes (Figures 5(c) and 5(d)) show higher LiF content than those in the 1m electrolytes (Figures 5(a) and 5(b)). And the samples at 60°C display higher LiF content compared with those at 27°C. These results indicate that higher salt concentration and elevated temperature can effectively induce more LiF content on the SEI layer. It was widely reported that LiF-rich SEI film could effectively protect Li metal and restrain lithium dendrite growth [6, 22]. The spectrum of C1s was analysed in [Supplementary-material supplementary-material-1]. There are four main peaks which attribute to CO_3_^2-^, CF_x_/C-SO_x_, C-O, and C-C/C-H, respectively [33, 34]. Compared to the samples at 27°C, those samples contain less carbon compounds. The function of SEI induced by temperature (60°C) and concentration (3m) has been demonstrated by additional deposition experiment. The Li-plating/-stripping process of the Li/Cu half cell (3m) was tested at 60°C for the first 15 cycles and then at a lower temperature of 27°C. As shown in [Supplementary-material supplementary-material-1], it is found that the well-formed SEI layer under the condition of 3m and 60°C helps increase the Coulombic efficiencies of Li-plating/-stripping during the following test at 27°C. As detected from [Supplementary-material supplementary-material-1], the surface of lithium deposition morphology is smoother than that sample cycling at 27°C without cycling at 60°C (Figure 4(c)). These results indicate that this SEI layer induced by the synergistic effects of high-salt concentration and elevated temperature can realize higher Coulombic efficiency and uniform lithium deposition in comparison with that sample at 27°C.

## 3. Discussion

Different deposited Li behaviours were detected with low concentration (1m) and high concentration (3m and 5m) electrolytes. High viscosity of the 5m electrolyte at 27°C-80°C hinders its practical application in the systematic investigation. And 80°C tends to aggravate the side reactions between Li metal and liquid electrolytes. As a consequence, we narrow the range of nine conditions and only investigate four conditions (1m and 3m electrolytes at 27°C and 60°C) in detail. A high concentration electrolyte theoretically increases the concentration of Li^+^ and TFSI^−^ in comparison to the 1m electrolyte. But high viscosity of concentrated electrolytes slows the mobility of both cations and anions, resulting in inhomogeneous ion distribution and large concentration polarization. Though rough morphology in the 3m electrolyte at room temperature is observed *via* in situ optical microscopy (Figure 3(c)), lithium dendrites still exist according to the ex situ SEM image (Figure 4(c)). The ionic conductivity of the 3m electrolyte at 60°C is a little bit higher than that of the 1m electrolyte at room temperature (27°C), which indicates that high viscosity of the concentrated electrolyte could be alleviated by raising the working temperature. In addition, raising the temperature will make uniform the distribution of Li^+^ and reduce the concentration polarization, owing to the accelerated mobility of ions at elevated temperature. Hence, high Li ion concentration and homogeneous anion distribution greatly decentralize the local charge density and impede the tip effect ([Supplementary-material supplementary-material-1]). A more uniform Li^+^ flux regulates the Li-plating/-stripping behaviour and realizes dendrite-free Li-metal anodes. Moreover, the LiF-rich SEI layer simultaneously suppresses the lithium dendrite and reduces side reactions (resulting in high CE).

In conclusion, we systematically investigate the synergistic effects of electrolyte concentration and elevated temperature on Li-plating/-stripping behaviour. In situ cells were designed to verify the optimized concentration (3 mol/kg) and temperature (60°C) for dendrite-free Li deposition by in situ optical microscopy. XPS depth profiles indicate that raising the temperature and increasing the salt concentration induce the formation of a LiF-rich SEI film, which further restrains dendrite growth and reduces side reactions. High average CE (99.2% over 150 cycles) and dendrite-free lithium deposition are achieved along with a high areal capacity of 2 mAh cm^−2^ on copper foil. This strategy shows a promising way towards reliable lithium metal anode for practical application.

## 4. Materials and Methods

### 4.1. Ionic Conductivity Test

The ionic conductivity of electrolytes was measured by AC impedance spectroscopy from 1 Hz to 1 MHz (BioLogic VSP-300 electrochemical workstation) in a symmetric cell (Pt|electrolyte|Pt).

### 4.2. Cyclic Voltammogram Test

BioLogic VSP-300 electrochemical workstation was used to obtain cyclic voltammograms of Li-plating/-stripping in different electrolytes by using a Cu foil disc (1.2 mm in diameter) as a working electrode and a Li-metal plate (1.2 mm in diameter) as reference and counter electrode. The scan rate was 0.5 mV s^−1^.

### 4.3. Li/Cu Half-Cell Test

The Cu foil was assembled with lithium metal as the counter electrode in the 2016-type coin cells. The electrolyte was 1, 3, and 5 mol/kg LiTFSI (lithium bis(trifluoromethanesulfonyl)imide) (Xiaoyuan, Shanghai, China) and 2 wt% LiNO_3_ (lithium nitrate) (Aladdin, China) in 1 : 1 (by volume) DOL (1,3-dioxolane) (Xiaoyuan, Shanghai, China) : DME (1,2-dimethoxyethane) (Xiaoyuan, Shanghai, China). The small current of 0.05 mA was set up at the first six cycles to remove the contaminants on the surface of electrodes and activate the batteries.

### 4.4. Symmetric Cell Test

Symmetric cells were tested by Li@Cu (Cu foil with 2 mAh cm^−2^ of prelithium) and lithium foil in the 2016-type coin cells. The current density and capacity were set as 0.5 mA cm^−2^ and 0.5 mAh cm^−2^.

### 4.5. Li@Cu/LiFePO_4_ Cells

LiFePO_4_ (MTI, Hefei, China) (LiFePO_4_ : carbon black conductive additive (Super P) : polyvinylidene fluoride (PVDF) = 7 : 2 : 1) on carbon-coated aluminum foil (~1.5 mg cm^−2^) and Cu foil with prelithium (2 mAh cm^−2^) were used as the cathode and anode, respectively, to fabricate the 2016-type coin cell. The electrolytes were 1 mol/kg and 3 mol/kg LiTFSI (lithium bis(trifluoromethanesulfonyl)imide) and 2 wt% LiNO_3_ (lithium nitrate) in 1 : 1 (by volume) DOL (1, 3-dioxolane) : DME (1, 2-dimethoxyethane).

### 4.6. Material Characterizations

Scanning electron microscopy (SEM) images were acquired by using a Nova NanoSem 450 instrument. A Thermo Fisher HAAKE MARSIII rotary rheometer was used to get the viscosity date of the electrolyte. X-ray photoelectron spectroscopy (XPS) was carried out to characterize the SEI layer of deposited Li by using a XSAM800 Ultra Spectrometer.

Materials and Methods should provide sufficient information to allow replication of the results.

## Supplementary Materials

Supplementary Materials Figure S1: the impedance results of symmetric cells in different concentration electrolytes. Figure S2: the voltage profiles for Li/Cu half cells. Figure S3: the chemical stability tests of Li metal in the 3m electrolyte at different working temperatures. Figure S4: the initial charge/discharge curves of the Li-plating/-stripping process in Li/Cu half cells. Figure S5: symmetric cells of Li/Li@Cu foil. Figure S6: cycling performance of Li@Cu/LiFePO4 cells at 1C. Figure S7: the schematic of in situ cells. Figure S8: the in situ optical images of the deposition morphology of Li in LB303 at 27°C. Figure S9: the in situ optical images of the explosive growth of Li dendrites in the 1m electrolyte at 27°C. Figure S10: the in situ optical images showed that Li dendrites grew up through the glass fibers. Figure S11: XPS results of C1s spectra of deposited Li. Figure S12: additional plating/stripping experiment with the different samples. Figure S13: illustration of Li deposition influenced by synergistic effects of high concentration electrolytes and working temperatures. Movie S1: the explosive growth of lithium dendrite at the edge of the 2D copper foil recorded by in situ optical microscope in the 1m electrolyte at 27°C. Movie S2: the explosive growth of lithium dendrite at the middle of the 2D copper foil recorded by in situ optical microscope in the 1m electrolyte at 27°C.
