# Supplementary material for: Synergistic Effects of Salt Concentration and Working Temperature towards Dendrite-Free Lithium Deposition
Source: Research (Wash D C). 2019 Nov 5;2019:7481319. doi: 10.34133/2019/7481319 (PMC6944514; doi:10.34133/2019/7481319)
Supplement: Supplementary Materials — Figure S1: the impedance results of symmetric cells in different concentration electrolytes. Figure S2: the voltage profiles for Li/Cu half cells. Figure S3: the chemical stability tests of Li metal in the 3m electrolyte at different working temperatures. Figure S4: the initial charge/discharge curves of the Li-plating/-stripping process in Li/Cu half cells. Figure S5: symmetric cells of Li/Li@Cu foil. Figure S6: cycling performance of Li@Cu/LiFePO4 cells at 1C. Figure S7: the schematic of in situ cells. Figure S8: the in situ optical images of the deposition morphology of Li in LB303 at 27°C. Figure S9: the in situ optical images of the explosive growth of Li dendrites in the 1m electrolyte at 27°C. Figure S10: the in situ optical images showed that Li dendrites grew up through the glass fibers. Figure S11: XPS results of C1s spectra of deposited Li. Figure S12: additional plating/stripping experiment with the different samples. Figure S13: illustration of Li deposition influenced by synergistic effects of high concentration electrolytes and working temperatures. Movie S1: the explosive growth of lithium dendrite at the edge of the 2D copper foil recorded by in situ optical microscope in the 1m electrolyte at 27°C. Movie S2: the explosive growth of lithium dendrite at the middle of the 2D copper foil recorded by in situ optical microscope in the 1m electrolyte at 27°C. [file 7481319.f1.zip › 7481319.f1/Supplementary Materials.docx]

Supplementary Materials for

**Synergistic Effects of Salt Concentration and Working Temperature towards Dendrite-free Lithium Deposition**

Panlong Li^1^, Chao Li^1^, Yang Yang^1^, Chanyuan Zhang^2^, Renhe Wang^1^, Yao Liu^1^,

Yonggang Wang^1^, Jiayan Luo^2^, Xiaoli Dong^1^*, Yongyao Xia^1^*

* Corresponding author. E-mail: [xldong@fudan.edu.cn](mailto:xldong@fudan.edu.cn), [yyxia@fudan.edu.cn](mailto:yyxia@fudan.edu.cn).

**This PDF file includes:**

- **Fig. S1.** The impedance results of Symmetric cells in different concentration electrolytes.
- **Fig. S2.** The voltage profiles for Li/Cu half cells.
- **Fig. S3.** The chemical stability tests of Li metal in 3m electrolyte at different working temperatures.
- **Fig. S4.** The initial charge/discharge curves of Li plating/stripping process in Li/Cu half cells.
- **Fig. S5.** Symmetric cells of Li/Li@Cu foil.
- **Fig. S6.** Cycling performance of Li@Cu/LiFePO_4_ cells at 1C.
- **Fig. S7.** The schematic of in-situ cells.
- **Fig. S8.** The in-situ optical images of the deposition morphology of Li in LB303 at 27^o^C.
- **Fig. S9.** The in-situ optical images of the explosive growth of Li dendrites in 1m electrolyte at 27^o^C.
- **Fig. S10.** The in-situ optical images showed that Li dendrites grew up through the glass fibers.
- **Fig. S11.** XPS results of C1s spectra of deposited-Li.
- **Fig. S12.** Additional plating/stripping experiment with the different samples.
- **Fig. S13.** Illustration of Li deposition influenced by synergistic effects of high concentration electrolytes and working temperatures.

Caption for Movies S1 to S2:

**Movie S1.** The explosive growth of lithium dendrite at the edge of 2D copper foil recorded by in-situ optical microscope in 1m electrolyte at 27^o^C.

**Movie S2.** The explosive growth of lithium dendrite at the middle of 2D copper foil recorded by in-situ optical microscope in 1m electrolyte at 27^o^C.


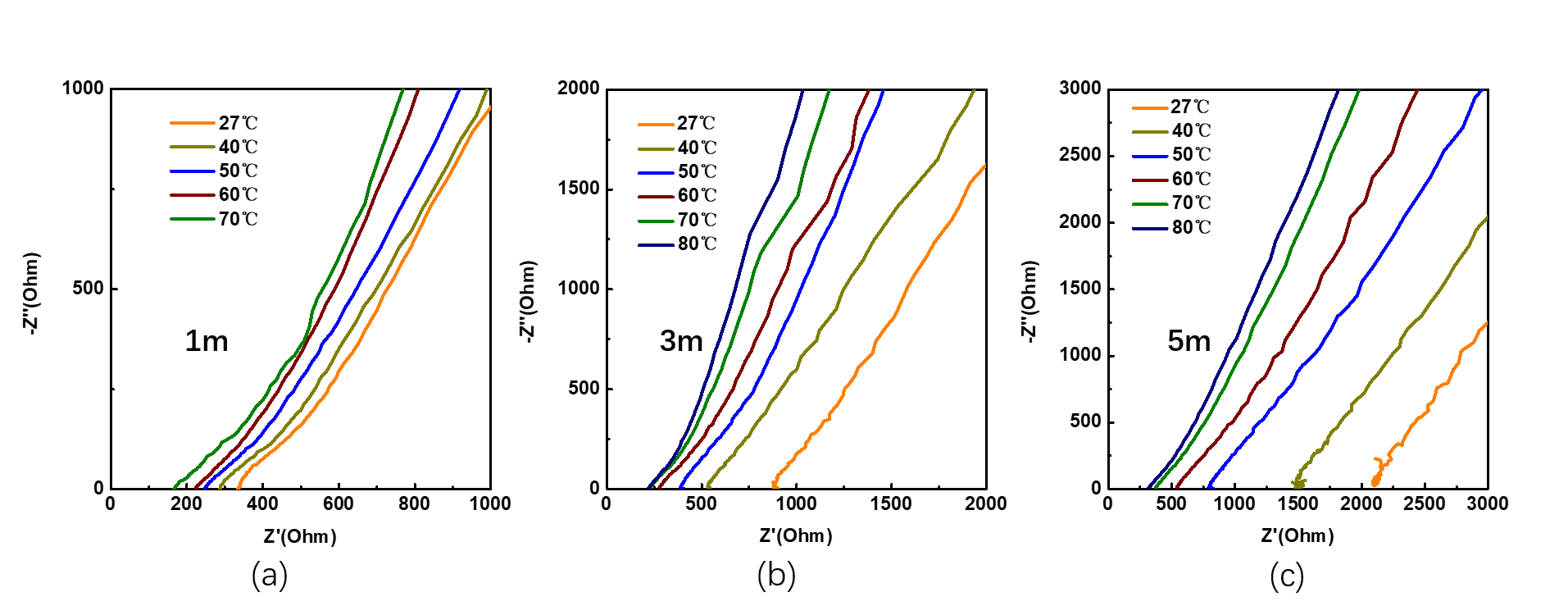


**Fig. S1. The impedance results of Symmetric cells in different concentration electrolytes.** (**a**) 1m, (**b**) 3m and (**c**) 5m electrolytes at different working temperatures.


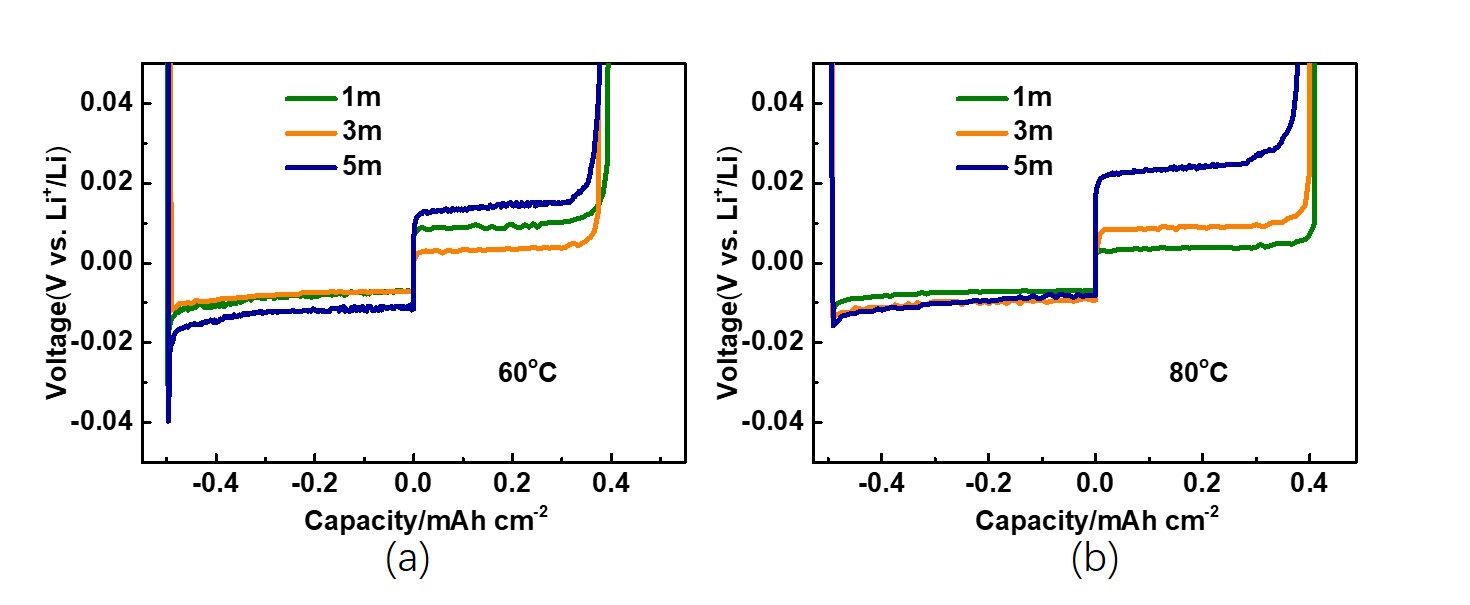


**Fig. S2. The voltage profiles for Li/Cu half cells.** The initial polarization curves of plating / stripping behavior of Li on copper foil at the current density of 0.5 mA cm^-2^ with a fixed capacity of 0.5 mA h cm^-2^ in 1m, 3m and 5m electrolyte at 60^o^C (a) and 80^o^C (b).


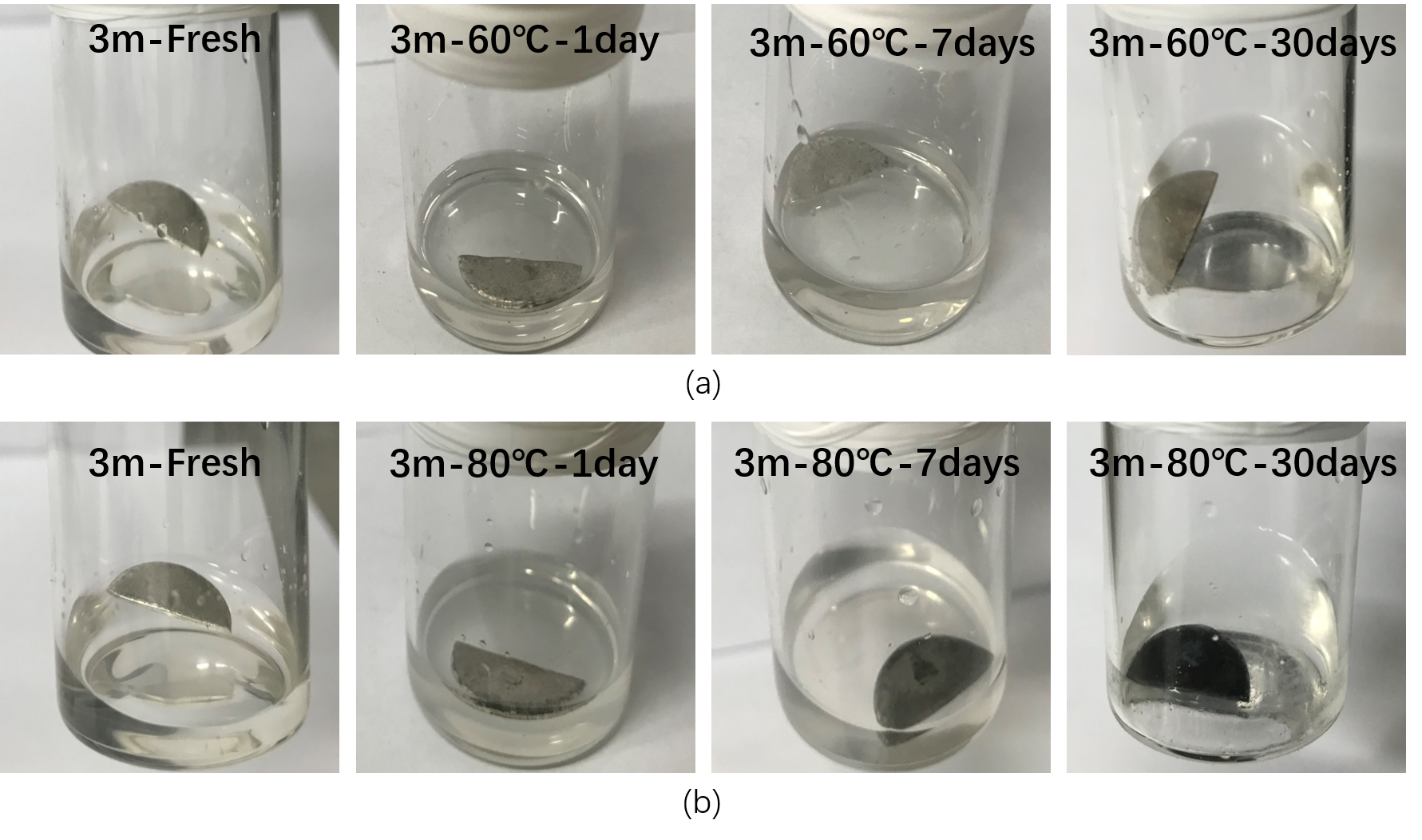


**Fig. S3. The chemical stability tests of Li metal in 3m electrolyte at different working temperatures.**


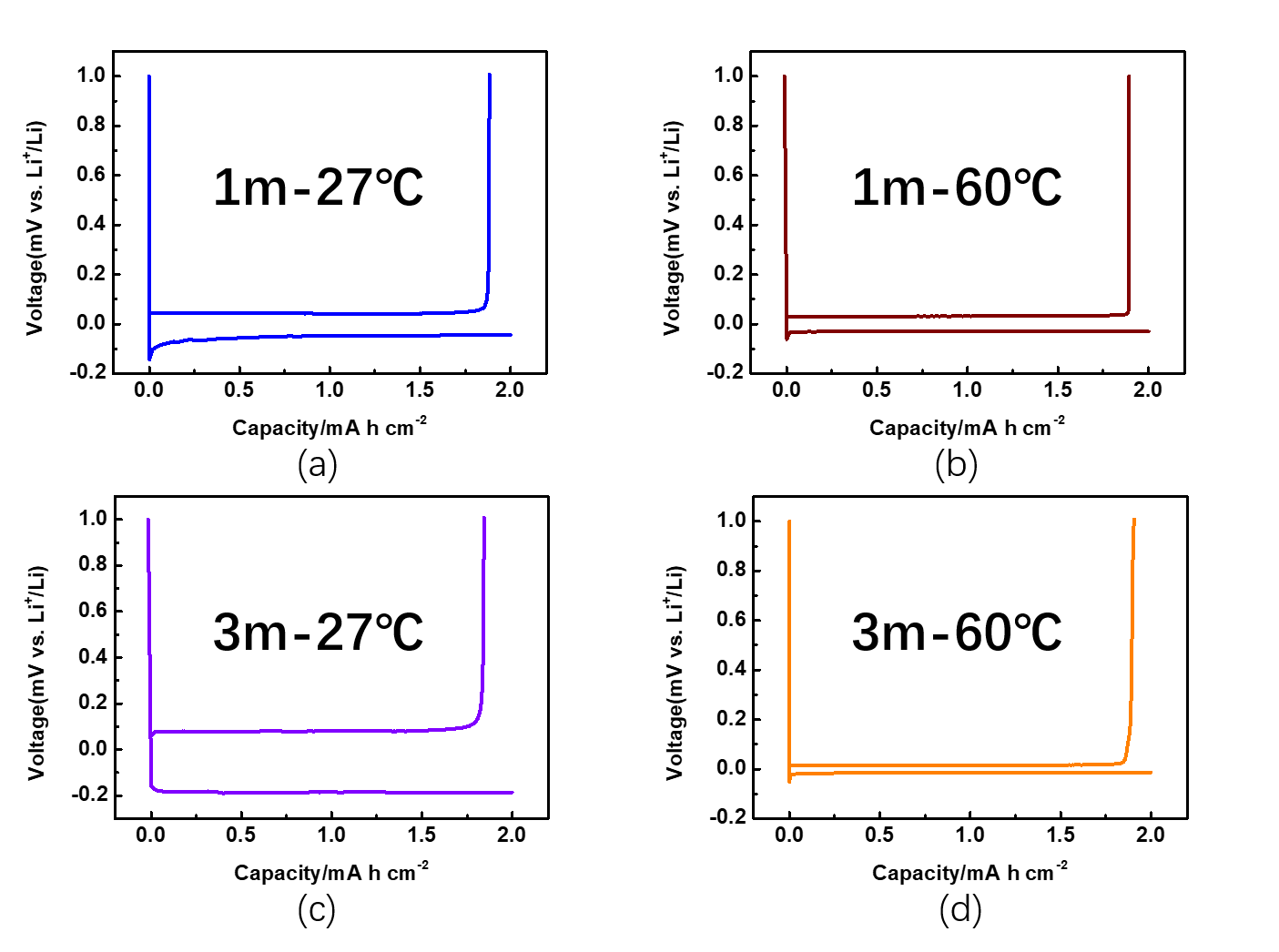


**Fig. S4. The initial charge/discharge curves of Li plating/stripping process in Li/Cu half cells.** A fixed capacity of 2.0 mA h cm^-2^ was plated/stripped on copper foil at the current density of 1 mA cm^-2^ in different concentration electrolytes and working temperatures. (**a**) 1m electrolyte at 27^o^C. (**b** 1m electrolyte at 60^o^C. (**c**) 3m electrolyte at 27^o^C. (**d**) 3m electrolyte at 60^o^C.


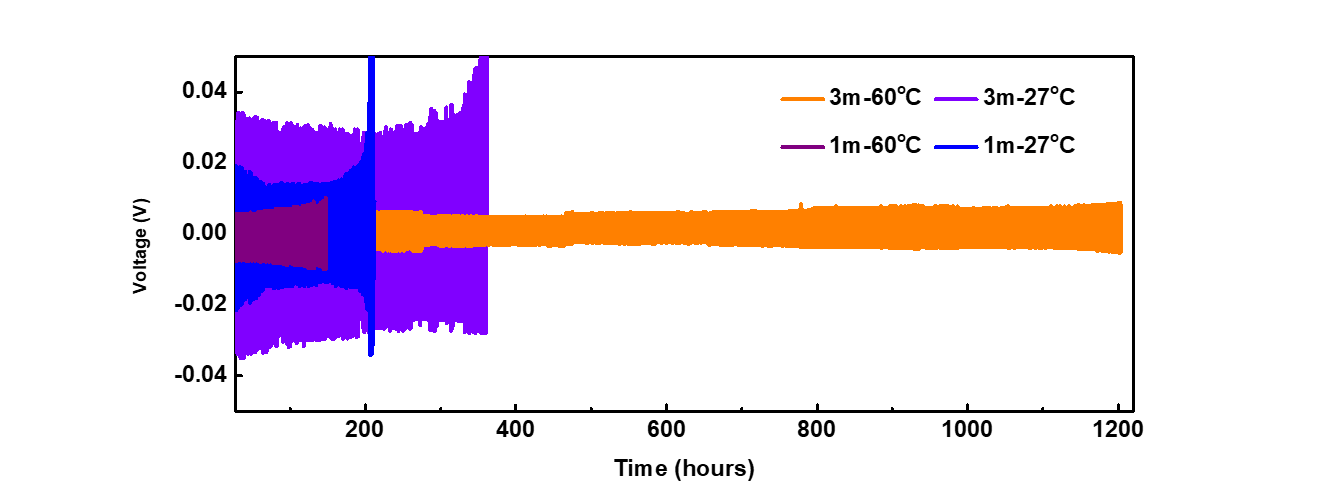


**Fig. S5. Symmetric cells of Li/Li@Cu foil.** (Cu foil electrodes with 2.0 mAh cm^-2^ of pre-deposition Li). The plating/stripping process was conducted at the current density of 0.5 mA cm^-2^ with a fixed capacity of 0.5 mA h cm^-2^ in 1m and 3m electrolytes at 27^o^C and 60^o^C.


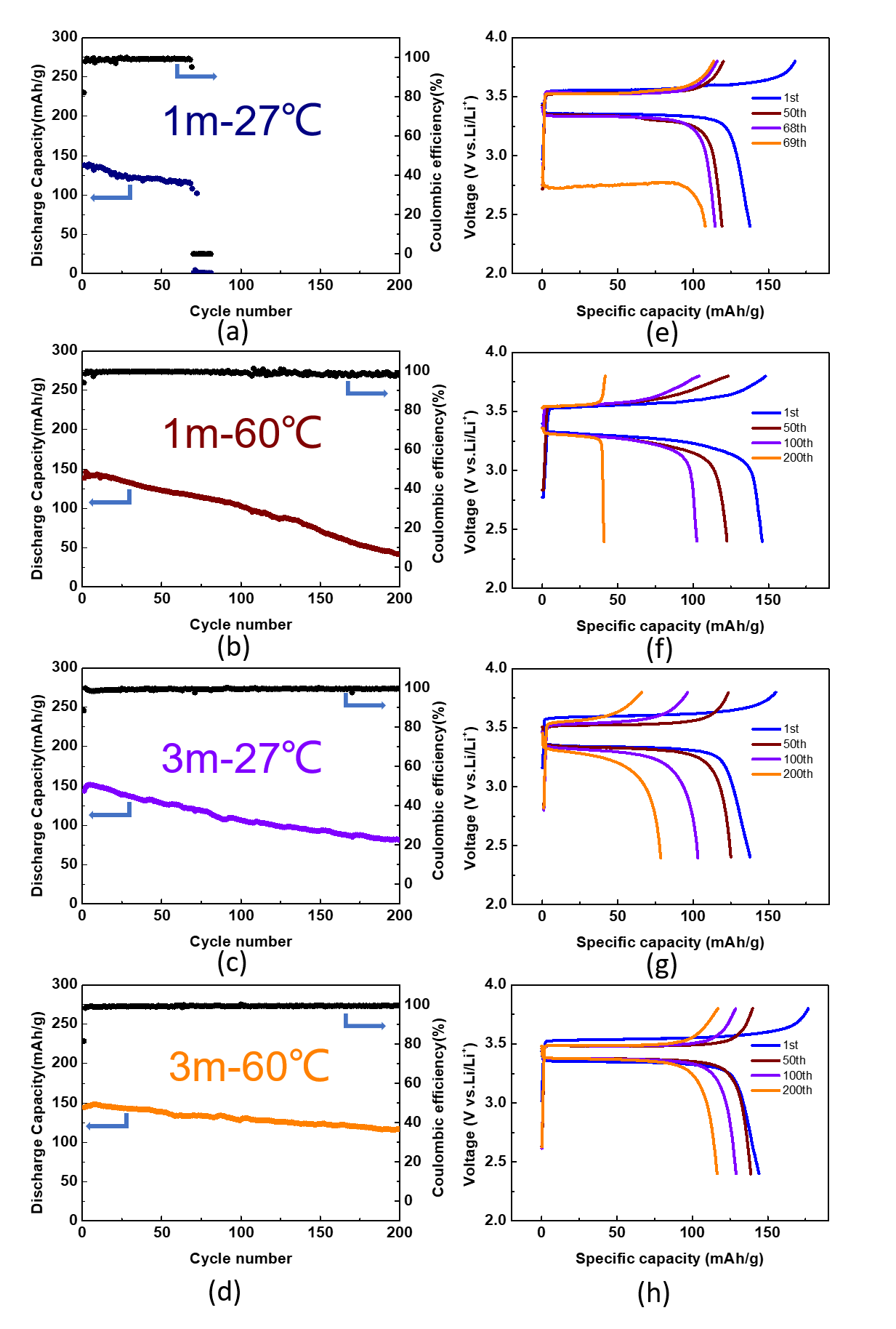


**Fig. S6.** **Cycling performance of Li@Cu/LiFePO_4_ cells at 1C.** (**a**)-(**d**) Cycling performance of Li@Cu/LiFePO_4_ at different temperatures and electrolytes. (**e**)-(**h**) The corresponding charge and discharge profiles of Li@Cu/LiFePO_4_ batteries.


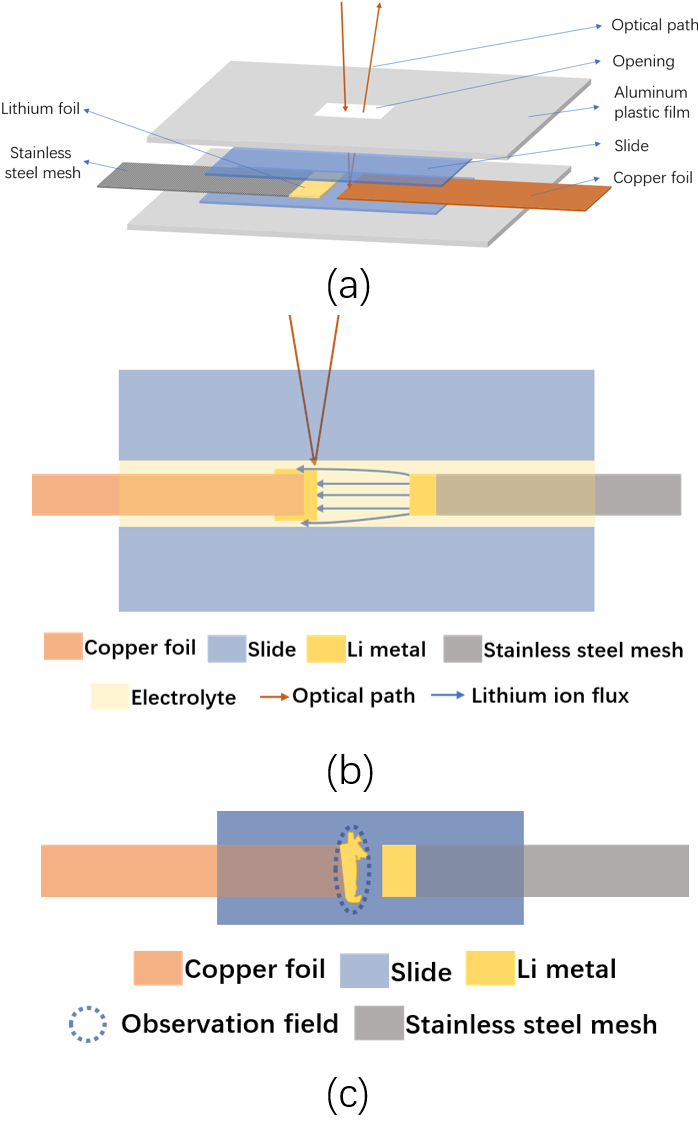


**Fig. S7. The schematic of in-situ cells.** (**a**) 3D schematic of the in-situ cells. (**b**) The side view of the in-situ cells. (**c**) The top view of the in-situ cells.


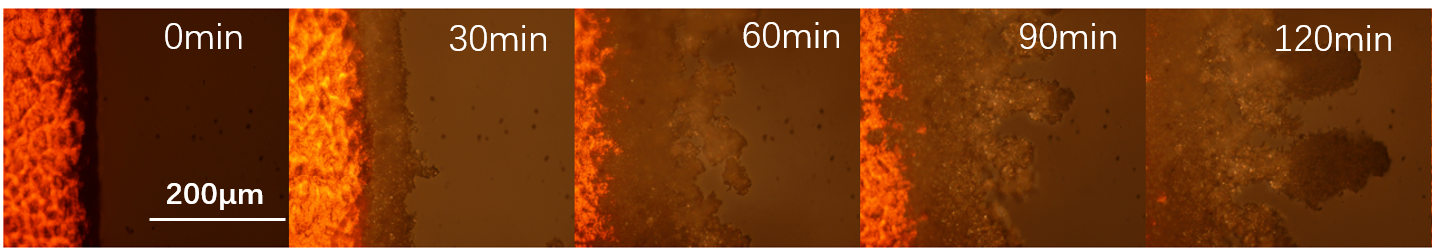


**Fig. S8.** **The in-situ optical images of the deposition morphology of Li in LB303 at 27oC.** (LB303, 1 M LiPF_6_ in EC/DEC/DMC = 1:1:1)


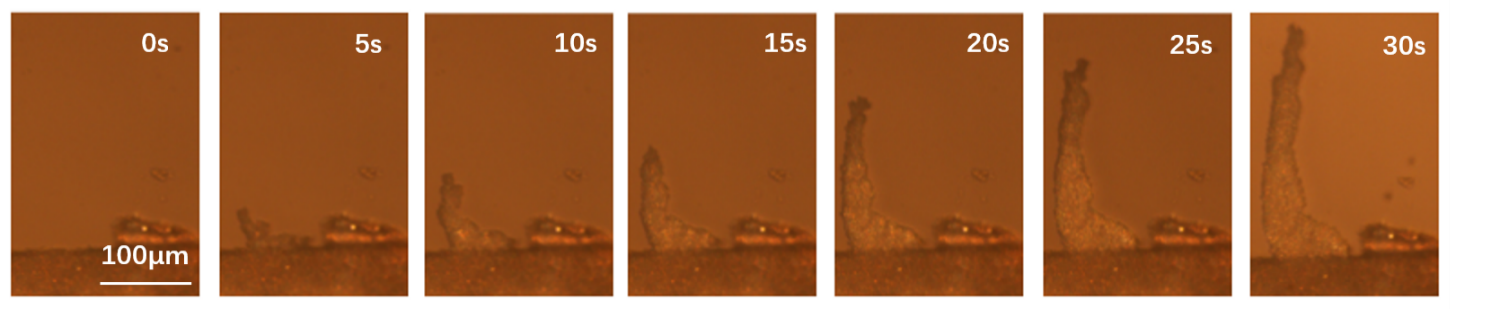


**Fig. S9.** **The in-situ optical images of the explosive growth of Li dendrites in 1m electrolyte at 27^o^C.**


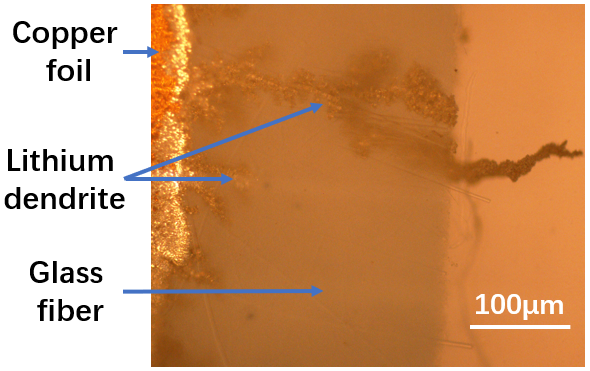


**Fig. S10.** **The in-situ optical images showed that Li dendrites grew up through the glass fibers.**


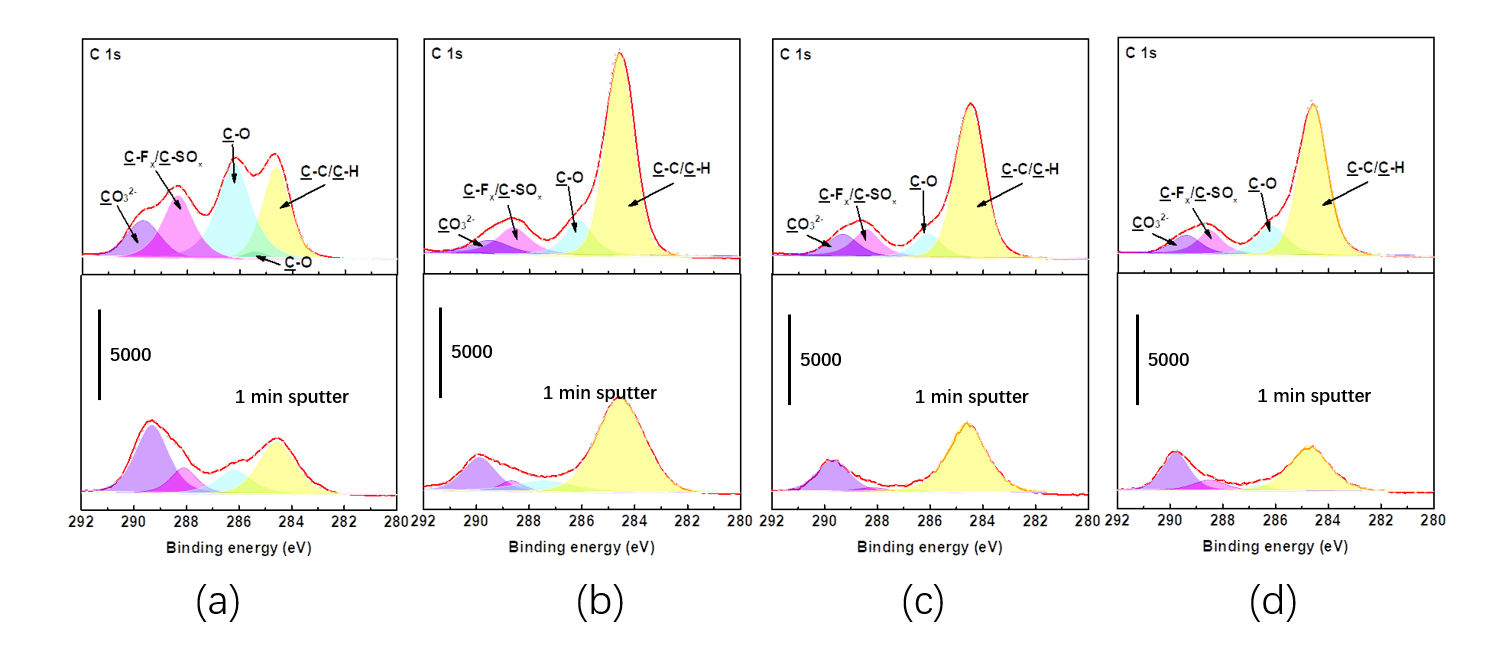


**Fig. S11. XPS results of C1s spectra of deposited-Li.** (**a**) C1s spectra of deposited-Li before and after 1 min Ar sputtering in 1m electrolyte at 27^o^C; (**b**) 1m electrolyte at 60^o^C; (**c**) 3m electrolyte at 27^o^C; (**d**) 3m electrolyte at 60^o^C.


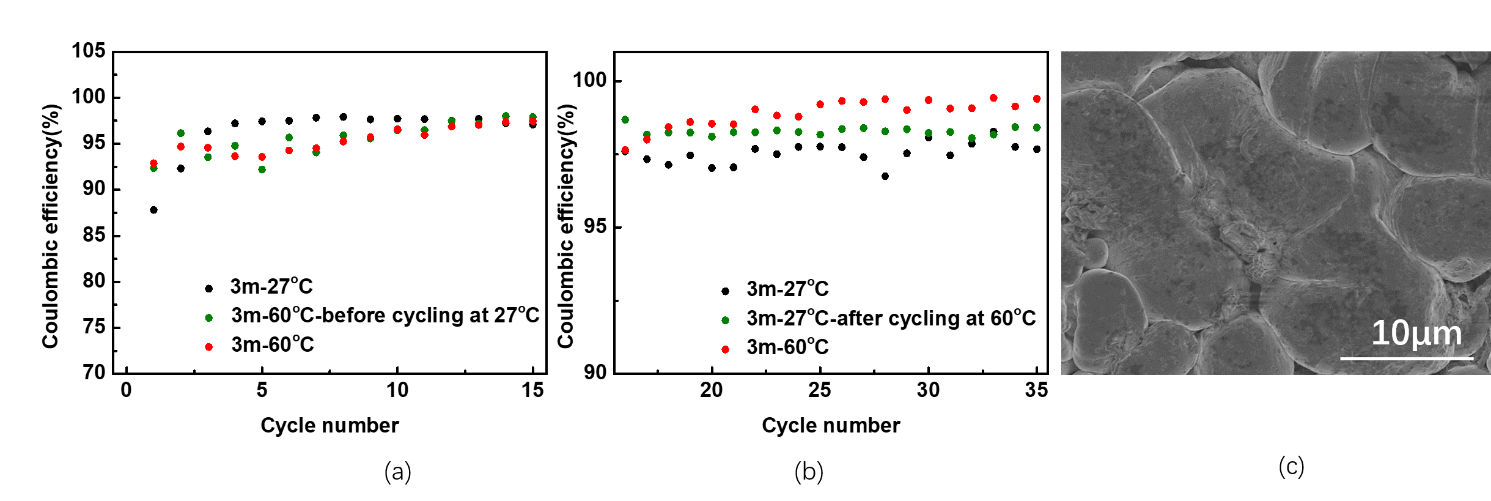


**Fig. S12. Additional plating/stripping experiment with the different samples** **at the current density of 1 mA cm^-2^ with a fixed capacity of 2 mA h cm^-2^ [Sample#1: in 3m electrolyte at 27^o^C; Sample#2: in 3m electrolyte at 60^o^C for first 15 cycles and at 27^o^C for following cycles; Sample#3: in 3m electrolyte at 60^o^C].** (**a**) Comparation of Coulombic efficiencies of Li-plating/stripping at different conditions. (b) Comparation of Coulombic efficiencies of Li-plating/stripping at different conditions. (c) The morphology of lithium deposition of the cell which was tested at 60^o^C then plating at 27^o^C.


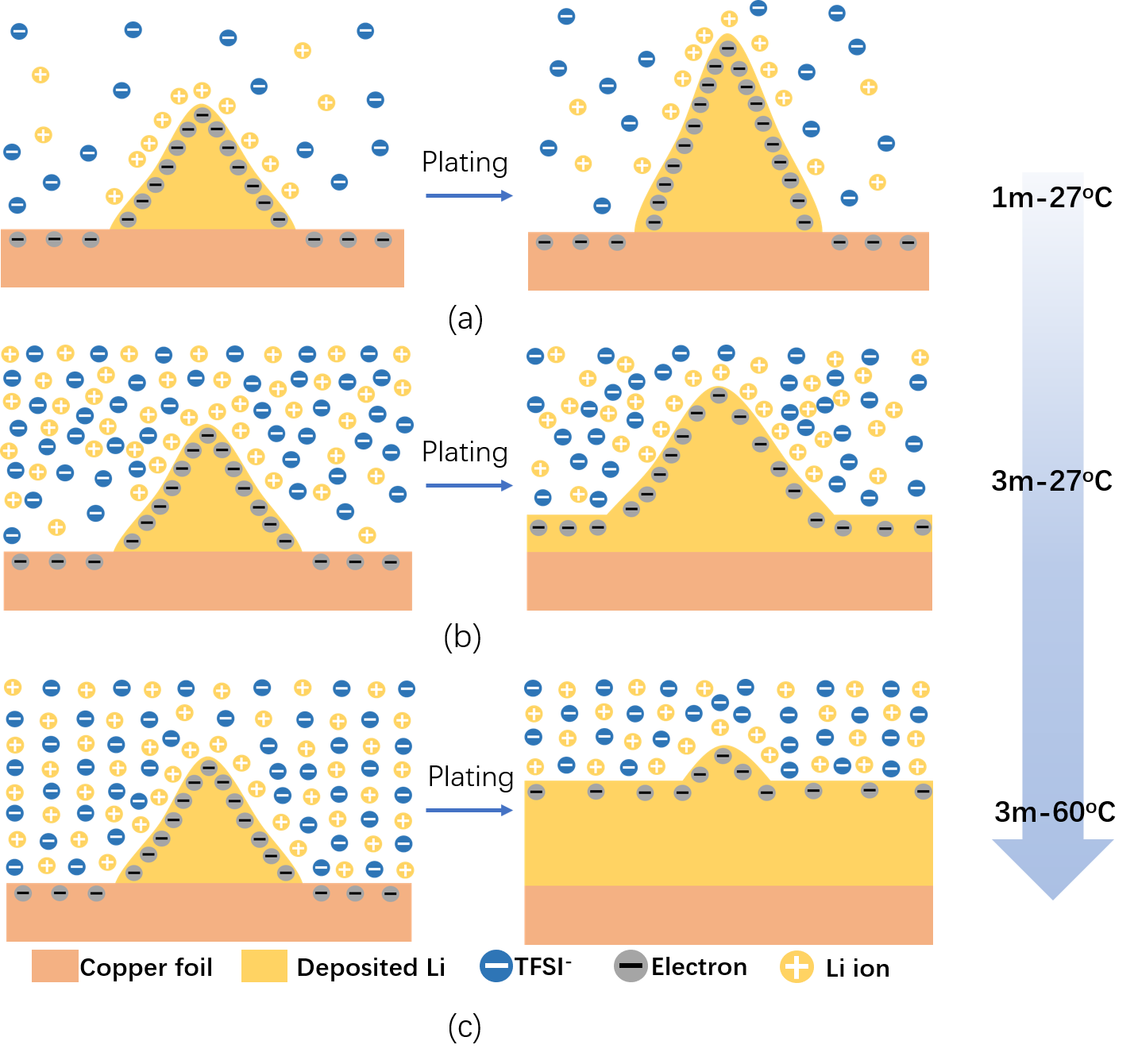


**Fig. S13. Illustration of Li deposition influenced by synergistic effects of high concentration electrolytes and working temperatures.** (**a**) Li dendrites grow up due to the tip effect in 1m electrolyte at 27^o^C. (**b**) In 3m electrolyte at 27^o^C, rising salt concentration can partly reduce the tip effect. However, non-uniform Li deposition cannot fully suppress the growth of lithium dendrites. (**c**) In 3m electrolyte at 60^o^C, rising temperature homogenizes the distribution of high-salt concentration ions, which decentralized the local charge density and reduced the tip effect. As a result, Li deposition becomes more uniform.
